# Challenges associated with insulin therapy progression among patients with type 2 diabetes: Latin American MOSAIc study baseline data

**DOI:** 10.1186/s13098-016-0157-1

**Published:** 2016-07-22

**Authors:** Bruno Linetzky, Brad Curtis, Gustavo Frechtel, Renan Montenegro, Miguel Escalante Pulido, Oded Stempa, Janaina Martins de Lana, Juan José Gagliardino

**Affiliations:** Eli Lilly and Company, Tronador 4890, Piso 12, CABA, C1430DNN Buenos Aires, Argentina; Eli Lilly and Company, Lilly Corporate Center, Indianapolis, IN 46285 USA; Servicio de Nutrición y Diabetes, Hospital Sirio Libanes, Campana 4658, C1419HN Buenos Aires, Argentina; School of Medicine of the Federal University of Ceará, Rua Capitao Francisco Pedro, 1290 Fortaleza, Ceara, 60430-370 Brazil; Hospital de Especialidades del Centro Médico de Occidente IMSS, Belisario Domínguez 1000, piso 2., Col. Independencia Guadalajara, Jalisco, Mexico; Eli Lilly and Company, Barranca del Muerto 329-1, Col. San José Insurgentes, Delegación Benito Juárez, Mexico, 03900 Distrito Federal Mexico; Eli Lilly and Company, Av. Morumbi 8.264, São Paulo, Brazil; CENEXA, Centro de Endocrinología Experimental y Aplicada (UNLP-CONICET La Plata), Calle 60 y 120, La Plata, Argentina

**Keywords:** Type 2 diabetes, Latin America, Observational study, Quality of care, Psychological impact, Diabetes knowledge, Diabetes self-care management, Insulin treatment, Diabetes education

## Abstract

**Background:**

Poor glycemic control in patients with type 2 diabetes is commonly recorded worldwide; Latin America (LA) is not an exception. Barriers to intensifying insulin therapy and which barriers are most likely to negatively impact outcomes are not completely known. The objective was to identify barriers to insulin progression in individuals with type 2 diabetes mellitus (T2DM) in LA countries (Mexico, Brazil, and Argentina).

**Methods:**

MOSAIc is a multinational, non-interventional, prospective, observational study aiming to identify the patient-, physician-, and healthcare-based factors affecting insulin intensification. Eligible patients were ≥18 years, had T2DM, and were treated with insulin for ≥3 months with/without oral antidiabetic drugs (OADs). Demographic, clinical, and psychosocial data were collected at baseline and regular intervals during the 24-month follow-up period. This paper however, focuses on baseline data analysis. The association between glycated hemoglobin (HbA1c) and selected covariates was assessed.

**Results:**

A trend toward a higher level of HbA1c was observed in the LA versus non-LA population (8.40 ± 2.79 versus 8.18 ± 2.28; p ≤ 0.069). Significant differences were observed in clinical parameters, treatment patterns, and patient-reported outcomes in LA compared with the rest of the cohorts and between Mexico, Brazil, and Argentina. Higher number of insulin injections and lower number of OADs were used, whereas a lower level of knowledge and a higher level of diabetes-related distress were reported in LA. Covariates associated with HbA1c levels included age (−0.0129; p < 0.0001), number of OADs (0.0835; p = 0.0264), higher education level (−0.2261; p = 0.0101), healthy diet (−0.0555; p = 0.0083), self-monitoring blood glucose (−0.0512; p = 0.0033), hurried communication style in the process of care (0.1295; p = 0.0208), number of insulin injections (0.1616; p = 0.0088), adherence (−0.1939; p ≤ 0.0104), and not filling insulin prescription due to associated cost (0.2651; p = 0.0198).

**Conclusion:**

MOSAIc baseline data showed that insulin intensification in LA is not optimal and identified several conditions that significantly affect attaining appropriate HbA1c values. Tailored public health strategies, including education, should be developed to overcome such barriers.

*Trial Registration* NCT01400971

## Background

Although it is widely accepted that tight glycemic control is associated with a decreased risk of diabetes-related complications [1–5], poor control (herein defined as HbA1c >7.0 %) is commonly recorded worldwide and the available data show that Latin America (LA) is not an exception [6–11]. Despite clear treatment algorithms established within international guidelines, insulin therapy is frequently delayed even after long periods of poor metabolic control [12, 13]. Furthermore, observational data and evidence provided by multiple clinical trials implemented in different countries demonstrate a lack of treatment goal achievement among insulin-treated patients [14–18].

Although insulin therapy has been shown to significantly reduce glycated hemoglobin (HbA1c) levels, patients and physicians are often reluctant to initiate insulin therapy. Studies suggest that the reasons for this inertia on behalf of patients include a perceived lack of efficacy, negative impact on lifestyle, injection phobia, and fear of weight gain or hypoglycemic events [19]. Physician barriers include fears for their patients’ safety (including weight gain and hypoglycemia), a perceived greater drain on physician’s resources (time and cost), and concern that insulin regimens are too complex for patients to understand and will result in poor adherence [20]. Health care system factors, such as limited access to medication, care, and out of pocket expenditures, represent additional barriers to insulin therapy initiation [21–23]. This multicomponent situation represents the major hurdles to overcome to achieve a successful initiation of, and persistence on, insulin therapy.

Despite these known barriers and their negative impact on the achievement of appropriate metabolic control, to the authors’ knowledge no longitudinal study is currently available that attempts to address this important issue. Moreover, considering the scarce achievement of treatment goals in patients under insulin treatment, it is necessary to identify the barriers to intensifying insulin therapy and which of these barriers are most likely to impact outcomes.

The Multinational Observational Study Assessing Insulin use (MOSAIc) study is a multinational observational cohort study aiming at identifying the patient-, physician-, and health care environment-based factors associated with insulin initiation and progression in patients with type 2 diabetes mellitus (T2DM) in real-world practice. Data collected include demographic, clinical, and psychosocial indicators at the patient and physician level and practice site characteristics recorded at baseline and regular intervals during a 24-month follow-up period [24]. This analysis attempts to identify particular challenges faced by patients treated with insulin in LA. We have compared baseline demographic, clinical, and psychosocial characteristics of the overall MOSAIc cohort to that of three LA countries.

## Methods

### Study design

The rationale and design of the MOSAIc study have been reported elsewhere [24]. Briefly, MOSAIc is a multinational, non-interventional, prospective, observational cohort study due for completion in December 2015. Participants were recruited from July 2011 to July 2013 at 223 sites in 18 countries [United Arab Emirates (UAE), Argentina, Brazil, Canada, China, Germany, India, Israel, Italy, Japan, Mexico, Russia, Saudi Arabia, South Korea, Spain, Turkey, the UK, and the US (including Puerto Rico)].

Study sites represented a combination of specialist and general practice centers in urban and rural areas. Participants were followed for 2 years after study enrollment, with visit windows approximately 6, 12, 18, and 24 months after the baseline visit, with such visits being part of their usual care.

The study was conducted following the ethical principles of the Helsinki Declaration, in accordance with good clinical practices and the applicable laws and regulations of the participant countries. The MOSAIc study was registered under ClinTrials.gov (NCT01400971). All patients completed informed consent forms approved by their country-specific institutional review boards (can be provided on request). The study’s analytic plan has been approved by the Brigham and Women’s Hospital Institutional Review Board.

### Study population

Inclusion criteria for participation in MOSAIc were age ≥18 years; diagnosis of T2DM; presentation to a study site as part of usual medical care; use of any commercially available initial insulin therapy for at least 3 months with or without any combination of approved non-insulin oral antidiabetic drugs (OADs) (e.g., metformin); and sufficient understanding of the primary language of the country to complete study surveys. Exclusion criteria were participation in another medical research study; use of intensive basal-bolus therapy (basal insulin in addition to three prandial doses); or initiation of insulin treatment with three daily injections of mixed insulin.

### Baseline data collection and patient-reported outcomes

Patient data for demographic and clinical characteristics, comorbid conditions, and insulin regimen were retrospectively collected (limited to 6 months before the baseline visit) from medical records at the study site.

Extensive information on patient-reported diabetes- and insulin-related knowledge, attitudes, hypoglycemia, general health behaviors, patient-provider relationship, and perceived physical and psychological well-being were collected at baseline using self-report questionnaires.

The Brief Diabetes Knowledge Test was used to evaluate patients’ understanding of their disease, such as how to manage insulin administration and how to treat hypoglycemia, with a summary score ranging from 0 (no questions correct) to 9 (all correct) [25].

The 17-item Diabetes Distress Scale was used to measure patients’ degree of concern about different aspects of their type 2 diabetes care and treatment, using a six-point Likert scale ranging from “Not a problem” to “A very serious problem” [26]. Mean items score and standard deviation (SD) are reported.

The Insulin Specific Adherence Questionnaire was used to evaluate adherence to insulin therapy and included a question to assess patients’ willingness to increase the frequency of injections. This question asked the participant to indicate to what extent he/she agreed with the statement: “I am willing to add additional injections to control my diabetes”.

The 25-item Interpersonal Processes of Care (IPC) survey measured how patients’ perceived the quality of their relationship with their providers over the past 12 months. Five alternative responses were provided for each question: 1 (never), 2 (rarely), 3 (sometimes), 4 (usually), and 5 (always). There are four positive IPC domains (elicited concerns, explained results, patient-centered decisions, and compassionate/respectful) in which higher scores correspond to better perceived interactions. Two IPC domains (hurried communication and discrimination) that were negatively framed in a way that better perceived interactions are represented by a lower score [27].

The Summary of Diabetes Self-Care Activities questionnaire was also administered in the study, analyzing three questions: “On how many of the last 7 days did you test your blood sugar the number of times recommended by your health-care provider?”, “How many of the last 7 days have you followed a healthful eating plan?”, and “On how many of the last 7 days did you participate in at least 30 min of physical activity?”. Responses ranged from 0 to 7 [28].

### Statistical analysis

Baseline participant characteristics were analyzed by region comparing LA participants with the rest of the cohort and by country comparing participants from Argentina, Brazil, and Mexico.

Categorical variables were described as the number and percentage of participants, and continuous variables were described using the mean and SD. Multiple imputation by Chained Equation was used to impute missing items [29]. Pooled analysis of variance (ANOVA) was used for continuous variables when comparing regional differences depending on whether the variables were imputed. Comparison of categorical variables was primarily undertaken using the Chi square test, except for insulin regimen where the Fisher’s exact test was used. The Cochran–Mantel–Haenszel test was used when comparing the number of oral agents. Pooled multivariate linear regression models were used to assess the association between HbA1c and selected covariates. For all statistical analyses, the significance level was set at ≤0.05. The imputation was done using Stata 13 (StataCorp LP; College Station, TX). All other analyses used SAS version 9.2 software (SAS Institute; Cary, NC).

## Results

A total of 4341 patients met all MOSAIc eligibility criteria and comprised the analyzed population; 521 were from LA (Argentina = 160; Brazil = 155; Mexico = 206). Demographic, clinical, and metabolic characteristics are listed in Table 1. Data were grouped as LA and non-LA participants, as well as by the three different LA countries. Comparable age values were recorded in all groups. Patients from Argentina were significantly older than those from the other two LA countries (p ≤ 0.0001).

**Table 1:** Demographic, clinical, and metabolic characteristics of the population by region and country

	All Mosaic cohort	LA	Non-LA countries	p	Argentina	Brazil	México	p
Mean age, years (SD)	61.77 (11.02)	61.99 (11.21)	61.74 (10.99)	0.6326	65.48 (10.55)	61.03 (9.51)	60.00 (12.26)	<0.0001
Gender—females, n (%)	2176 (50.1 %)	293 (56.2 %)	1883 (49.3 %)	0.0029	76 (47.5 %)	100 (64.5 %)	117 (56.8 %)	0.0095
Education								
Primary school, n (%)	1291 (29.7 %)	251 (48.2 %)	1040 (27.2 %)	<0.0001	83 (51.9 %)	65 (41.9 %)	103 (50.0 %)	0.0474
High school or more, n (%)	2715 (62.5 %)	235 (45.1 %)	2480 (64.9 %)		69 (43.1 %)	69 (44.5 %)	97 (47.1 %)	
Insurance								
Private, n (%)	917 (21.1 %)	134 (25.7 %)	783 (20.5 %)	0.0223	56 (35.0 %)	48 (31.0 %)	30 (14.6 %)	<0.0001
Public, n (%)	2229 (51.3 %)	247 (47.4 %)	1982 (51.9 %)		65 (40.6 %)	76 (49.0 %)	106 (51.5 %)	
Uninsured, n (%)	848 (19.5 %)	103 (19.8 %)	745 (19.5 %)		35 (21.9 %)	16 (10.3 %)	52 (25.2 %)	
Mean diabetes duration, years (SD)	12.65 (7.98)	13.52 (8.77)	12.54 (7.87)	0.0083	13.71 (9.77)	13.46 (7.78)	13.42 (8.69)	0.9492
Comorbidities								
MI or CAD, n (%)	824 (19.0 %)	38 (7.3 %)	786 (20.6 %)	<0.0001	16 (10.0 %)	15 (9.7 %)	7 (3.4 %)	0.0217
Stroke, n (%)	151 (3.5 %)	10 (1.9 %)	141 (3.7 %)	0.0384	4 (2.5 %)	5 (3.2 %)	1 (0.5 %)	0.1393
Congestive heart failure, n (%)	237 (5.5 %)	6 (1.2 %)	231 (6.0 %)	<0.0001	0 (0.0 %)	3 (1.9 %)	3 (1.5 %)	0.2383
Nephropathy, n (%)	685 (15.8 %)	48 (9.2 %)	637 (16.7 %)	<0.0001	14 (8.8 %)	17 (11.0 %)	17 (8.3 %)	0.6574
Neuropathy, n (%)	1194 (27.5 %)	85 (16.3 %)	1109 (29.0 %)	<0.0001	14 (8.8 %)	22 (14.2 %)	49 (23.8 %)	0.0004
Retinopathy, n (%)	954 (22.0 %)	78 (15.0 %)	876 (22.9 %)	<0.0001	24 (15.0 %)	24 (15.5 %)	30 (14.6 %)	0.9709
Depression, n (%)	370 (8.5 %)	46 (8.8 %)	324 (8.5 %)	0.7899	4 (2.5 %)	16 (10.3 %)	26 (12.6 %)	0.0024
Hypertension, n (%)	2994 (69.0 %)	335 (64.3 %)	2659 (69.6 %)	0.0140	112 (70.0 %)	110 (71.0 %)	113 (54.9 %)	0.0013
Hyperlipidemia, n (%)	2484 (57.2 %)	259 (49.7 %)	2225 (58.2 %)	0.0002	81 (50.6 %)	93 (60.0 %)	85 (41.3 %)	0.0019
HbA1c, mean (SD)	8.20 (2.47)	8.40 (2.79)	8.18 (2.28)	0.0686	8.08 (2.05)	8.34 (2.38)	8.70 (3.55)	0.1108
HbA1c physician reported goal (SD)	7.02 (0.77)	7.10 (0.76)	7.01 (0.73)	0.011	7.17 (0.65)	7.11 (0.89)	7.04 (0.79)	0.2917
BMI, mean (SD)	29.58 (6.39)	29.78 (5.64)	29.55 (6.49)	0.4437	31.24 (6.22)	30.21 (5.46)	28.32 (4.81)	<0.0001
Systolic blood pressure, mean (SD)	132.42 (16.83)	132.94 (17.24)	132.34 (16.72)	0.4395	133.64 (13.69)	135.49 (19.88)	130.49 (17.54)	0.0214

*BMI* body mass index, *CAD* coronary artery disease, *LA* = Latin America, *MI* myocardial infarction, *SD* standard deviation

The LA region had a higher percentage of female participants (56.2 %) compared to the global population, particularly in Brazil (64.5 %). Similarly, a significantly higher rate of participants with an education level of primary school or lower was also recorded in LA compared to non-LA countries (48.2 versus 27.2 %), particularly in Argentina (51.9 %) and Brazil (50.0 %). There was also a significant difference comparing the percentage of people with health insurance, with the lowest figures recorded in Mexico (25.2 %). The LA population had a longer duration of diabetes than the overall MOSAIc cohort, with no significant difference among the three LA countries. Conversely, the rate of comorbidities (associated cardiovascular risk factors, microvascular complications, and macrovascular events) was lower in the LA population.

Baseline HbA1c levels were above the treatment targets recommended by international guidelines, with no significant differences among all the groups, although lower levels were recorded in the non-LA population (8.40 ± 2.79 versus 8.18 ± 2.28; p ≤ 0.069). Among LA countries, higher but not significantly different HbA1c values were recorded in Mexico (8.70 ± 3.55).

There were no significant differences between participants classified as overweight from LA or non-LA countries; conversely, there were significant differences among those classified as obese among countries, with the highest and lowest rates recorded in Argentina and Mexico, respectively (p ≤ 0.0001).

Systolic blood pressure values were close to target values recommended by international guidelines, with Brazil and Mexico having the highest and lowest values, respectively (p = 0.0214).

Treatment patterns varied across countries included in the study (Table 2). A higher number of daily insulin injections were reported in LA compared to non-LA countries, with Argentina having significantly more reported insulin injections compared to Brazil and Mexico (p ≤ 0.0001 for both). Basal insulin alone was more frequently used in LA than in the rest of the MOSAIc cohort, with the highest rate recorded in Brazil among LA countries (74.8 %; p ≤ 0.0001). A higher percentage of LA participants also required basal insulin more than once per day. Important differences were also recorded in the use of concomitant OADs agents between the LA and non-LA population, as well as within LA countries (p ≤ 0.0001 for both). Metformin was the most commonly utilized therapy, with the highest and lowest figures recorded in Brazil and Mexico, respectively (p ≤ 0.0001).

**Table 2:** Treatment patterns by region and country

	All Mosaic cohort	LA	Non-LA countries	p	Argentina	Brazil	México	p
Freq of insulin injections/day mean	1.63 (0.68)	1.80 (0.68)	1.60 (0.67)	<0.0001	1.99 (0.76)	1.73 (0.73)	1.69 (0.54)	<0.0001
Insulin regimen								
Basal insulin only								
Overall, n (%)	2168 (49.9 %)	365 (70.1 %)	1803 (47.2 %)	<0.0001	103 (64.4 %)	116 (74.8 %)	146 (70.9 %)	<0.0001
Once, n (%)	1656 (76.4 %)	152 (41.6 %)	1504 (83.4 %)		37 (35.9 %)	52 (44.8 %)	63 (43.2 %)	
More than once, n (%)	512 (23.6 %)	213 (58.4 %)	299 (16.6 %)		66 (64.1 %)	64 (55.2 %)	83 (56.8 %)	
Mixed insulin only								
Overall, n (%)	1284 (29.6 %)	70 (13.4 %)	1214 (31.8 %)		36 (22.5 %)	0 (0.0 %)	34 (16.5 %)	
Once, n (%)	112 (8.7 %)	4 (5.7 %)	108 (8.9 %)		1 (2.8 %)	0 (0.0 %)	3 (8.8 %)	
More than once, n (%)	1172 (91.3 %)	66 (94.3 %)	1106 (91.1 %)		35 (97.2 %)	0 (0.0 %)	31 (91.2 %)	
Short acting only								
Overall, n (%)	170 (3.9 %)	11 (2.1 %)	159 (4.2 %)		1 (0.6 %)	1 (0.6 %)	9 (4.4 %)	
Once, n (%)	37 (21.8 %)	2 (18.2 %)	35 (22.0 %)		1 (100.0 %)	0 (0.0 %)	1 (11.1 %)	
More than once, n (%)	133 (78.2 %)	9 (81.8 %)	124 (78.0 %)		0 (0.0 %)	1 (100.0 %)	8 (88.9 %)	
Other insulin combinations								
Overall, n (%)	597 (13.8 %)	64 (12.3 %)	533 (14.0 %)		20 (12.5 %)	27 (17.4 %)	17 (8.3 %)	
Once, n (%)	172 (28.8 %)	14 (21.9 %)	158 (29.6 %)		2 (10.0 %)	9 (33.3 %)	3 (17.6 %)	
More than once, n (%)	425 (71.2 %)	50 (78.1 %)	375 (70.4)		18 (90.0 %)	18 (66.7 %)	14 (82.4 %)	
Other antidiabetic medication								
No. of OADs, mean (SD)	1.22 (1.09)	0.84 (0.88)	1.27 (1.10)	<0.0001	0.66 (0.68)	1.33 (0.91)	0.61 (0.84)	<0.0001
Metformin, n (%)	2437 (56.1 %)	280 (53.7 %)	2157 (56.5 %)	0.2400	82 (51.3 %)	122 (78.7 %)	76 (36.9 %)	<0.0001
Sulfonylurea, n (%)	1389 (32.0 %)	92 (17.7 %)	1297 (34.0 %)	<0.0001	13 (8.1 %)	55 (35.5 %)	24 (11.7 %)	<0.0001
Dipeptidyl peptidase-4 inhibitor, n (%)	538 (12.4 %)	38 (7.3 %)	500 (13.1 %)	0.0002	2 (1.3 %)	20 (12.9 %)	16 (7.8 %)	0.0003
Alpha-glucosidase inhibitor, n (%)	321 (7.4 %)	7 (1.3 %)	314 (8.2 %)	<0.0001	1 (0.6 %)	2 (1.3 %)	4 (1.9 %)	0.5536
GLP-1, n (%)	143 (3.3 %)	5 (1.0 %)	138 (3.6 %)	0.0015	2 (1.3 %)	2 (1.3 %)	1 (0.5 %)	0.6678
Other drug, n (%)	443 (10.2 %)	14 (2.7 %)	429 (11.2 %)	<0.0001	5 (3.1 %)	5 (3.2 %)	4 (1.9 %)	0.6952

*GLP1* Glucagon-like peptide-1, *LA* Latin America, *OADs* oral antidiabetic drugs, *SD* standard deviation

Individual Diabetes Knowledge scores were low in the overall MOSAIc population, with lower figures in the LA versus non-LA countries (4.16 ± 2.23 versus 4.89 ± 2.19; p < 0.0001). The lowest figures were recorded in Mexico (3.93 ± 2.10) and Brazil (3.88 ± 1.91) (p = 0.0002) (Table 3).

**Table 3:** Self-reported outcomes by region and country

	All Mosaic cohort	LA	Non-LA countries	p	Argentina	Brazil	México	p
Diabetes knowledge, mean (SD)	4.80 (2.26)	4.16 (2.23)	4.89 (2.19)	<0.0001	4.72 (2.30)	3.88 (1.91)	3.93 (2.10)	0.0002
DDS total, mean (SD)	2.27 (1.14)	2.49 (1.32)	2.24 (1.11)	<0.0001	2.17 (1.19)	3.14 (1.36)	2.26 (1.21)	<0.0001
Self-care activities								
Self-monitoring, mean (SD)	3.60 (2.62)	3.70 (2.68)	3.58 (2.64)	0.3238	4.89 (2.52)	3.30 (2.64)	3.09 (2.52)	<0.0001
General diet, mean (SD)	4.44 (2.24)	4.57 (2.21)	4.42 (2.24	0.1576	4.86 (2.02)	3.89 (2.49)	4.85 (2.11)	<0.0001
Exercise mean (SD)	2.86 (2.44)	2.42 (2.40)	2.92 (2.43)	<0.0001	2.14 (2.32)	1.93 (2.35)	3.01 (2.37)	<0.0001
Adherence (does not miss shots), n (%)	3290 (75.8 %)	398 (76.4 %)	2892 (75.7 %)	0.7793	134 (83.8 %)	104 (67.1 %)	160 (77.7 %)	0.0622
Willingness to add additional injection, n (%)	2383 (54.9 %)	351 (67.4 %)	2032 (53.2 %)	<0.0001	99 (61.9 %)	107 (69.0 %)	145 (70.4 %)	0.1973
Not fill in insulin prescription due to cost, n (%)	460 (10.6 %)	53 (10.2 %)	407 (10.7 %)	0.5832	6 (3.8 %)	14 (9.0 %)	33 (16.0 %)	0.0004
IPC								
Hurried communication, mean (SD)	1.57 (0.70)	1.53 (0.77)	1.58 (0.69)	0.1184	1.25 (0.42)	1.69 (0.80)	1.62 (0.88)	<0.0001
Elicited concerns, mean (SD)	3.92 (1.05)	3.73 (1.18)	3.95 (1.03)	<0.0001	4.15 (0.91)	3.51 (1.17)	3.56 (1.25)	<0.0001
Explained results-medications, mean (SD)	3.92 (1.02)	4.03 (1.04)	3.91 (1.01)	0.0110	4.41 (0.68)	3.84 (1.07)	3.87 (1.17)	<0.0001
Patient-centered decision making, mean (SD)	3.37 (1.22)	3.30 (1.35)	3.37 (1.20)	0.2293	3.74 (1.15)	2.87 (1.20)	3.29 (1.47)	<0.0001
Compassionate, respectful, mean (SD)	4.10 (0.89)	4.16 (0.97)	4.10 (0.87)	0.1189	4.53 (0.61)	4.12 (0.99)	3.90 (1.11)	<0.0001
Discriminated, mean (SD)	1.50 (0.73)	1.43 (0.67)	1.51 (0.73)	0.0123	1.45 (0.69)	1.37 (0.68)	1.46 (0.66)	0.4117

*DDS* Diabetes Distress Scale, *IPC* Interpersonal Processes of Care, *LA* Latin America

A small but statistically significant difference was observed in the patients’ Diabetes Distress Scale scores between LA and the rest of the study population (p ≤ 0.0001); an important and significant difference was also observed among LA countries, with highest level of distress recorded in Brazil (3.14 ± 1.36) and the lowest in Argentina (2.17 ± 1.19) (p ≤ 0.0001) (Table 3).

The summary of self-care activities questionnaire showed a lower number of days with at least 30 min of physical activity reported among study participants in LA. Comparison of the number of days performing self-monitoring activities among the three LA countries showed higher values in Argentina, higher number of days following a healthy diet in Argentina and Brazil, and more days with physical activity practices in Mexico (Table 3).

A similar level of adherence was reported in LA compared to the rest of the MOSAIc participants, but a trend to a lower level of adherence was reported in Brazil (67.1 %). LA patients expressed more willingness to add additional injections to control their diabetes (67.4 versus 53.2 %; p < 0.0001).

Although no significant difference in the rate of not filling the prescription due to cost was observed between LA and the rest of the MOSAIc cohort, important variations were observed at the country level, with the lowest and highest rates in Argentina and Mexico, respectively (p = 0.0004).

Differences in the nature of the reported patient-health care provider relationship are depicted in Table 3. Lower levels of “hurried communication” were reported in Argentina, as well as higher scores in the domains of “elicited concerns”, “explained results”, “compassionate and respectful style”, and “patient centered decision making”, compared to the other LA countries.

Table 4 shows the analysis of variables associated with HbA1c levels. After the adjustment for potential confounders, patients in LA countries had similar levels of HbA1c compared to the rest of the MOSAIc cohort. The variables significantly associated with HbA1c levels were age (−0.0129; p < 0.0001), number of other OADs (0.0835; p = 0.0264), having higher education level (−0.2261; p = 0.0101), following a healthy diet (−0.0555; p = 0.0083), self-monitoring blood glucose (−0.0512; p = 0.0033), a hurried communication style in the interpersonal process of care questionnaire (0.1295; p = 0.0208), the number of insulin injections (0.1616; p = 0.0088), being adherent to the insulin treatment (−0.1939; p = 0.0104), and no insulin prescription adherence due to associated cost (0.2651; p = 0.0198).

**Table 4:** Variables associated with HBA1c levels (univariate and multivariate analysis)

	Unadjusted regression			Adjusted regression		
	Estimate	95 % CI	p	Estimate	95 % CI	p value
Age	−0.0206	(−0.03, −0.02)	<0.0001	−0.0129	(−0.02, −0.01)	0.0001
Gender—female	0.1085	(−0.01, 0.23)	0.0735	0.0589	(−0.07, 0.19)	0.3632
Diabetes duration	−0.0054	(−0.01, 0.00)	0.1866	0.0036	(−0.01, 0.01)	0.4219
BMI	0.0114	(−0.00, 0.02)	0.0643	0.0075	(−0.00, 0.02)	0.2243
Number of OAD	0.0631	(−0.00, 0.13)	0.0639	0.0835	(0.01, 0.16)	0.0264
Insulin-mixed only	0.1715	(0.03, 0.31)	0.0143	0.0402	(−0.14, 0.22)	0.6625
Short acting only	0.4457	(0.11, 0.78)	0.0098	0.3583	(−0.00, 0.72)	0.0506
Other	0.2045	(0.02, 0.39)	0.0323	0.0892	(−0.11, 0.29)	0.3717
Country group—LA	0.2248	(−0.01, 0.46)	0.0620	0.2129	(−0.05, 0.48)	0.1077
Education level—high school	−0.1189	(−0.28, 0.04)	0.1496	−0.1436	(−0.32, 0.03)	0.1010
College	−0.1936	(−0.36, −0.03)	0.0211	−0.2261	(−0.40, −0.06)	0.0101
Insurance status—public	−0.2764	(−0.46, −0.09)	0.0037	−0.1834	(−0.38, 0.02)	0.0700
Private	−0.2186	(−0.48, 0.04)	0.0974	−0.1407	(−0.40, 0.12)	0.2788
SC—general diet	−0.0758	(−0.12, −0.04)	0.0004	−0.0555	(−0.10, −0.02)	0.0083
Specific diet	−0.0329	(−0.08, 0.01)	0.1451	−0.0368	(−0.08, 0.01)	0.1025
Exercise	−0.0413	(−0.07, −0.01)	0.0035	−0.0266	(−0.06, 0.00)	0.0798
Blood Glucose testing	−0.0658	(−0.09, −0.04)	<0.0001	−0.0512	(−0.08, −0.02)	0.0033
IPC-hurried communication	0.1800	(0.08, 0.28)	0.0004	0.1295	(0.02, 0.24)	0.0208
Elicited concerns	0.0145	(−0.08, 0.11)	0.7506	0.0414	(−0.05, 0.13)	0.3745
Explained results	−0.0424	(−0.15, 0.06)	0.4186	−0.0038	(−0.11, 0.10)	0.9422
Patient-centered decision	0.0251	(−0.06, 0.11)	0.5659	0.0513	(−0.03, 0.14)	0.2323
Compassionate/respectful	−0.0346	(−0.14, 0.07)	0.5215	−0.0109	(−0.12, 0.10)	0.8461
Discriminated style	−0.0039	(−0.11, 0.11)	0.9434	−0.0818	(−0.19, 0.03)	0.1403
DDS-total distress	0.1682	(0.11, 0.23)	<0.0001	0.0660	(−0.00, 0.14)	0.0655
Insulin injection frequency	0.2001	(0.10, 0.30)	0.0002	0.1616	(0.04, 0.28)	0.0088
Adherence (no missed shots)	−0.4575	(−0.60, −0.32)	<0.0001	−0.1939	(−0.34, −0.05)	0.0104

*BMI* body mass index, *CI* confidence interval, *DDS* Diabetes Distress Scale, *IPC* Interpersonal Process of Care, *LA* Latin America, *OADs* oral antidiabetic drugs, *SC* self-care

## Discussion

The current analysis of MOSAIc study baseline data provides relevant information regarding the potential challenges that individuals with T2DM face when using insulin in LA countries. Although people from the three LA countries included in the study share some of these challenges with the whole cohort, others appear to be more specific for the region. These findings highlight, from a public health perspective, the importance of implementing more locally tailored solutions to optimize blood glucose control in individuals with T2DM treated with insulin.

A common problem recorded was the poor degree of metabolic control (HbA1c ≈ 8 %), that coincides with data reported consistently in previous studies [7, 9, 10, 30].

This poor metabolic control was observed despite the wide variety of treatment patterns recorded in the studied population; in fact, patients in the three LA countries have a different treatment pattern compared to other regions, namely, a higher rate of basal insulin use and a lower rate of OADs agents used. Conversely, a comparable rate of metformin prescription was recorded in LA and non-LA countries. However, metformin was differently prescribed in LA countries, with a higher rate in Brazil (78.7 %) and a lower rate in Mexico (37 %). The recommendation of Asociación Latinoamericana de Diabetes (ALAD) guidelines regarding the use of metformin and precaution with the use of sulfonylureas may explain, at least partly, such a prescription pattern [31].

The low rate of incretin therapies use is also noteworthy, despite data showing that they are associated with a better HbA1c control and a lower risk of hypoglycemia and weight gain compared to insulin treated patients [32–34]. Clearly, none of the variety of treatment alternatives employed were effective in attaining the HbA1c target values recommended by international guidelines to prevent development and progression of chronic complications.

The linear regression analysis identified many variables associated with attainment of HbA1c treatment goals, with some of them unmodifiable (such as the age of the patients). Similar results have been reported in the ABCs of good management study in China [35, 36].

Other variables identified were the number of other associated OADs, the number of insulin injections, and adherence to insulin treatment, demonstrating once again that adherence is a key factor in attaining treatment targets whereas treatment complexity negatively affects long-term adherence and increases hospitalization rates [37]. The cost of treatment was also identified as a potential barrier to attaining Hb1c target values, which was confirmed by several other studies [23, 38].

Other factors affecting the attainment of HbA1c target values included level of education, healthy diet, performance of self-monitoring blood glucose, and a hurried communication style in the interpersonal process of care. Certainly, all of them have a common denominator: education.

Several authors have shown, in many populations, that educational programs using cognitive reframing are associated with improved outcomes [39–41]. Furthermore, Brownson et al. also reported that self-management programs for T2DM implemented at the primary care level were cost-effective from the perspective of a healthcare system when considering cost savings as a result of reductions in long-term complications [42].

In LA, we have shown that the implementation of a structured education program for individuals with T2DM (PEDNID-LA, Programa de Educación de Diabéticos No Insulinodependientes en América Latina) significantly improved the clinical and metabolic parameters that were tested and decreased the cost of treatment by 64 % [39]. More recently, the 3-year prospective education study implemented in the province of Corrientes (Argentina; PRODIACOR, PROgrama DIAbetes CORrientes), demonstrated similar clinical, metabolic, and psychological improvements [43]. This study also showed that education, regardless of the method used, is an effective tool to improve the care and outcomes of those with T2DM. However, the combined education of patients and physicians provided the greatest and most consistent and sustained clinical and metabolic improvement at the best drug treatment cost-effective ratio [43].

Similarly, a long-term multi-center education trial implemented in Italy by Trento and colleagues showed that healthcare behaviors, clinical and metabolic indicators, and quality of life were significantly better in the intervention group than in the control group [44].

Our study has several limitations, mainly associated with the nature of observational research (i.e., observational studies cannot provide causal evidence of an effect, in our case the real impact of conditioning factors on attainment of HbA1c target values). Baseline data were not available for all patients for all variables considered, thus we used multiple imputation with chained equations, a well-recognized method that accommodates both categorical and continuous variables, to impute missing values. Although this approach assumes that the missing values are missed at random, it is not possible to prove this assumption. Consequently, we also used a complete case analysis approach and results were quantitatively similar. Finally, the demographic, clinical, and psychosocial characteristics of the enrolled patients may be different from those individuals with T2DM in the general population of each country (24); this last bias could be of a lower magnitude because we recruited patients from both endocrinology and primary care practice sites with different practice locations (urban/rural), sizes, and practice types (academic/stand-alone) to maximize the data generalizability.

## Conclusions

The MOSAIc baseline data showed that patients under an initial scheme of insulin treatment in LA and non-LA countries are not achieving appropriate glycemic control, and this analysis identified several conditions that significantly affect the attainment of HbA1c values suggested by international guidelines. Appropriate glycemic control can effectively prevent the development and progression of chronic complications that decrease quality of life and increase cost of care over time. Although some of these factors are not modifiable (e.g., age), most of them can be significantly removed by educational strategies. Therefore, policy makers, particularly in the LA region where health resources are frequently scarce, might seriously consider the wide implementation of educational activities to improve the metabolic control of individuals with diabetes. This strategy could effectively decrease the heavy burden of the disease on health budget, the society, and particularly on individuals with diabetes.

## Abbreviations

ALAD: Asociación Latinoamericana de Diabetes
ANOVA: analysis of variance
HbA1c: glycated hemoglobin
IPC: Interpersonal Processes of Care
LA: Latin America
MOSAIc: Multinational Observational Study Assessing Insulin use
OADs: oral antidiabetic drug
PEDNID-LA: Programa de Educación de Diabéticos No Insulinodependientes en América Latina
Prodiacor: PROgrama DIAbetes CORrientes
SD: standard deviation
T2DM: type 2 diabetes mellitus
UAE: United Arab Emirates
